# Differential Effects of Statins on Inflammatory Interleukin-8 and Antimicrobial Peptide Human Β-Defensin 2 Responses in *Salmonella*-Infected Intestinal Epithelial Cells

**DOI:** 10.3390/ijms19061650

**Published:** 2018-06-02

**Authors:** Fu-Chen Huang, Shun-Chen Huang

**Affiliations:** 1 Department of Pediatrics, Kaohsiung Chang Gung Memorial Hospital and Chang Gung University College of Medicine, Kaohsiung 833, Taiwan; 2 Department of Pathology, Kaohsiung Chang Gung Memorial Hospital, Kaohsiung 833, Taiwan; shuang@cgmh.org.tw

**Keywords:** *Salmonella*, statins, innate immunity, intestinal epithelia, cytokines, antimicrobial peptide

## Abstract

Alternative therapies are needed to reduce the use of antibiotics and incidence of drug-resistant *Salmonellosis*. Previous studies have revealed important roles of statins in regulating innate immunity. Therefore, we investigated the effects of statins on innate immunity in *Salmonella*-infected intestinal epithelial cells (IECs), which are involved in mucosal innate immunity. SW480 cells and Akt siRNA- or vitamin D receptor (VDR) siRNA-transfected SW480 cells were infected by wild-type *S.* Typhimurium strain SL1344 in the presence or absence of statins. The mRNA or protein expression was analyzed by real-time quantitative PCR or western blot analysis, respectively. Simvastatin or fluvastatin caused IL-8 (interleukin-8) suppression, but increased hBD-2 mRNA expression in *Salmonella*-infected SW480 cells. Both statins enhanced phosphorylated Akt and VDR expressions. Akt or VDR knockdown by siRNA counteracted the suppressive effect of simvastatin on IL-8 expression, whereas VDR knockdown diminished the enhanced hBD-2 expression in *Salmonella*-infected SW480 cells. Therefore, we observed differential regulation of statins on inflammatory IL-8 and anti-microbial hBD-2 expressions in *Salmonella*-infected IECs via PI3K/Akt signaling and VDR protein expression, respectively. The enhanced activity of antimicrobial peptides by statins in *Salmonella*-infected IECs could protect the host against infection, and modulation of pro-inflammatory responses could prevent the detrimental effects of overwhelming inflammation in the host.

## 1. Introduction

The incidence of food-borne human *Salmonella enterica* serotype *enteritidis* infections has increased substantially. Antibiotic resistance among some serotypes of clinical *Salmonella* isolates has been increasing, even though multi-drug resistance (resistance to three or more classes of antibiotics) in *Salmonella* remained steady in the United States [1], with similar trends being reported from Europe and Taiwan [2].

Intestinal epithelial cells (IECs) act not only as a barrier to gut pathogens, but as an essential element of the mucosal innate immune system by the secretion of inflammatory cytokines, chemokines, and antimicrobial peptides following infection with *Salmonella*. Antimicrobial peptides, such as human β–defensins-2 (hBD-2), are secreted to kill the microorganisms at mucosal surfaces, while chemokines, such as interleukin-8 (IL-8), promote the transmigration of basolateral neutrophils to the intestinal lumen and induction of diarrhea, and give rise to the characteristic pathology of colitis [3].

Antimicrobial therapies, including microbicidal and microbiostatic therapies, are powerful tools for the treatment of several infectious diseases. Antibiotics are a type of antimicrobial drugs used in the treatment and prevention of bacterial infections. However, the increased resistance of microorganisms to currently used antibiotics has generated the need for alternative agents with potential antimicrobial activity.

Statins are cholesterol-lowering drugs targeting HMG-CoA reductase, thereby reducing the risk of coronary disorders and hypercholesterolemia. The beneficial effects of statins beyond cholesterol lowering have been explored. A growing body of evidence from both clinical trials and basic science studies suggest that statins have anti-inflammatory properties, which may additionally lead to clinical efficacy. Statin therapy might be associated with a lower incidence of infection-related morbidity and mortality. Patients receiving statin therapy may experience reduced infection-associated mortality due to pneumonia [4], bloodstream infection [5], sepsis [6], or multiple organ dysfunction syndrome [7]. Clinically, the use of statins may provide protection against *Clostridium difficile-*associated diarrhea [8] as suggested in a retrospective case–control study conducted in hospitalized patients. Simvastatin decreases IL-6 and IL-8 production in human oral epithelial cells [9]. Statins also result in decreased lipopolysaccharide-induced secretion of IL-6 and TNF-α by monocytes and macrophages [10]. However, the mechanism behind the antimicrobial activity of statins and how statins protect against lethal bacteremia, colitis, and sepsis remains inconclusive.

We have previously demonstrated that phosphoinositide-3 kinases (PI3Ks)/Akt have an anti-inflammatory effect on interleukin-8 (IL-8) production in *Salmonella*-infected IECs [11], and NOD2 was reported to mediate human β-defensin-2 (hBD-2) induction in epithelial cells [12]. Vitamin D receptor (VDR), an intracellular transcription factor, conducts the expression of unique groups of genes that orchestrate the distinct and cell-specific biological activities of its ligands. NOD2 is one of the VDR target genes [13]. Therefore, we aim to investigate the effects of statins on hBD-2 and IL-8 expressions in *Salmonella*-infected IECs as well as the signaling pathways and proteins that are involved.

## 2. Results and Discussion

### 2.1. Statins Suppressed IL-8 mRNA and Enhanced hBD-2 mRNA Expression in Salmonella-Infected SW480 Cells

**Figure 1:**
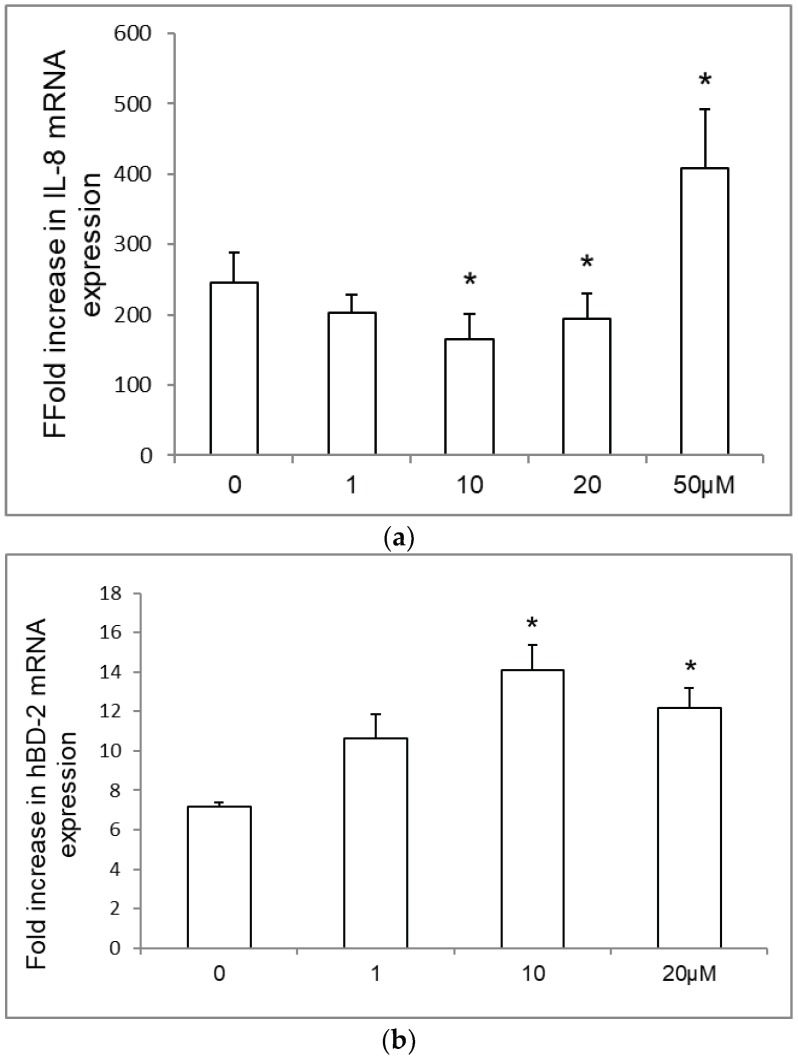
Effect of statins on IL-8 and hBD-2 mRNA expressions in *Salmonella*-infected SW480 cells. Cultured SW480 cells were uninfected or infected for 1 h with *S.* Typhimurium wild-type strain SL1344 (SL) in the presence or absence of different simvastatin concentrations (1, 10, 20, 50 μM). Total RNA extracted from the cultured cells after infection was transcribed to cDNA and analyzed by real-time quantitative PCR to estimate the IL-8 and hBD-2 mRNA expressions. The expressions of IL-8 (**a**) and hBD-2 mRNAs (**b**) were normalized to the corresponding GAPDH expression and are shown as fold increases over the uninfected control cells. The results indicate the means and standard deviations for duplicate wells from at least three separate experiments (* *p* < 0.05 compared to *Salmonella* infection only).

We examined the effect of statins on IL-8 and hBD-2 mRNA expressions in *Salmonella*-infected IECs. Intestinal epithelial SW480 cells were either uninfected or infected with wild-type *S.* Typhimurium strain SL1344 for one hour in the presence or absence of different simvastatin concentrations. Real-time quantitative PCR (RT-PCR) was performed to measure IL-8 and hBD-2 mRNA expressions. In Figure 1, we demonstrated that SL1344 infection induced IL-8 and hBD-2 mRNA expressions (normalized to GAPDH) in SW480 cells after a one-hour infection. Simvastatin suppressed IL-8 mRNA expression in *Salmonella*-infected SW480 cells when its concentration was increased to 10 and 20 μM, whereas it enhanced hBD-2 mRNA expression. In contrast, *Salmonella*-induced IL-8 mRNA expression was enhanced by a high concentration (50 μM) of simvastatin.

### 2.2. Involvement of Akt Signaling in the Simvastatin-Mediated Negative Regulation of IL-8 Expression in Salmonella-Infected SW480 Cells

**Figure 2:**
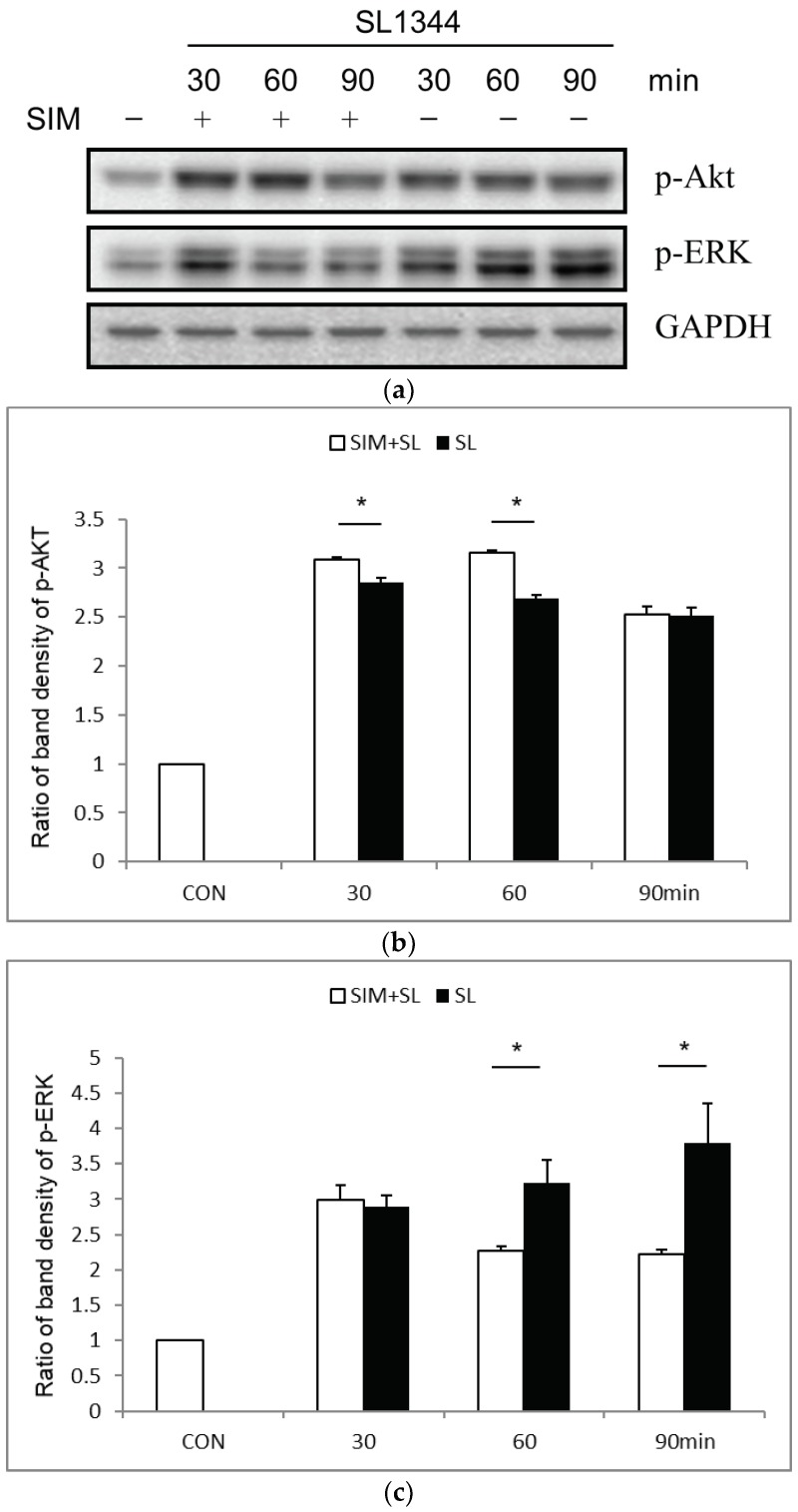

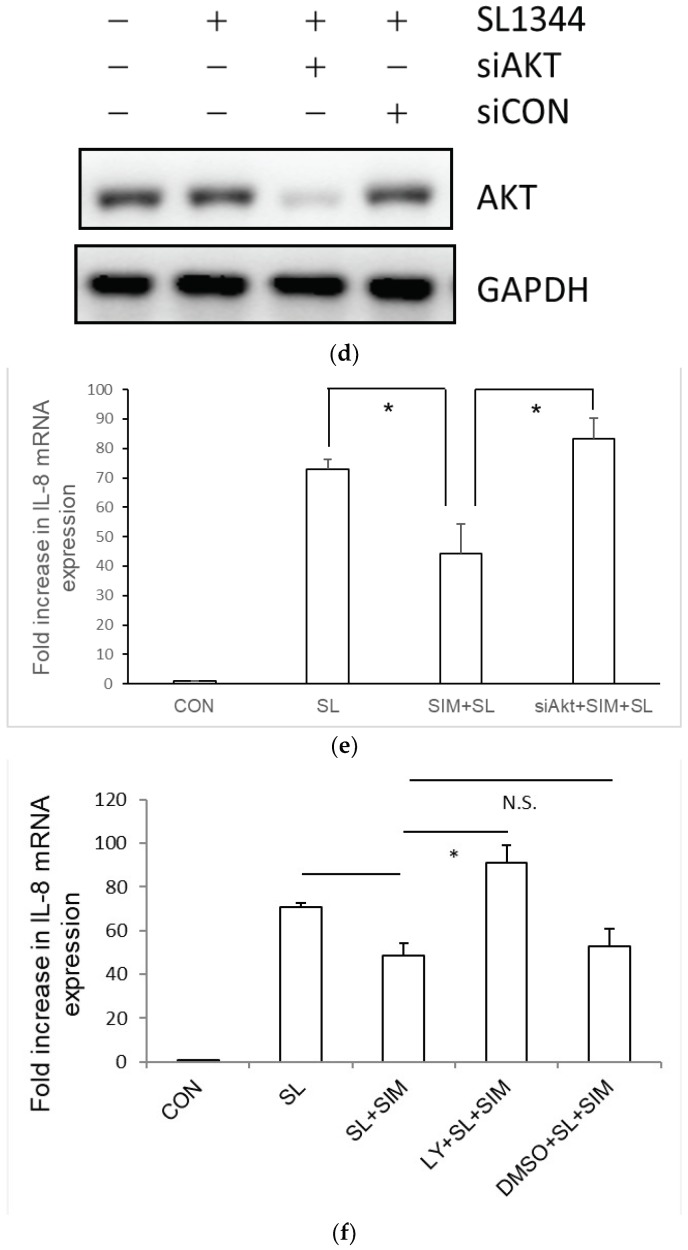

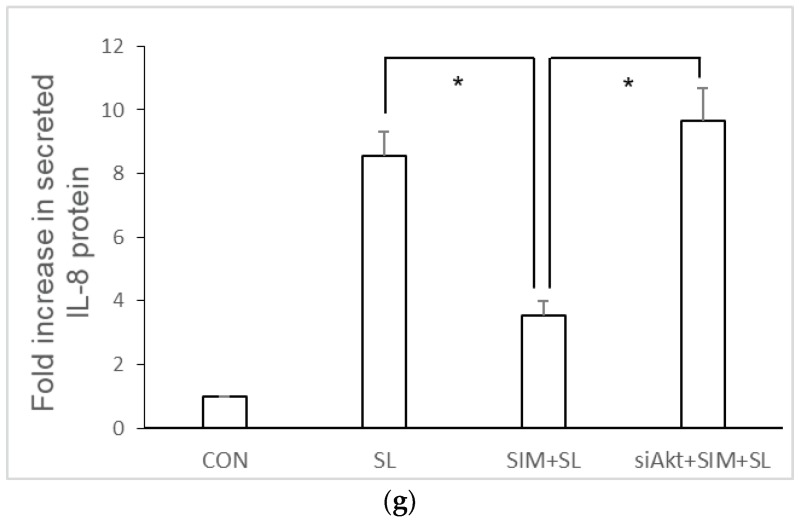
Involvement of Akt signaling pathways in the simvastatin-mediated suppression of IL-8 expression in *Salmonella*-infected SW480 cells. SW480 cells were transfected with control and Akt siRNAs (siAkt = siRNA against Akt) for 48 h. The untransfected or transfected cells were left uninfected or infected with the wild-type *S.* Typhimurium strain SL1344 (SL) for one hour in the presence or absence of 10 μM simvastatin (SIM) or PI3K inhibitor LY294002 (LY). The activation of Akt and ERK was analyzed in whole cell proteins by western blotting with antibodies to phosphorylated (p) Akt and ERK. Representative immunoblots (**a**) and densitometric quantification of the immunoreactive bands of phosphorylated Akt (p-Akt) (**b**) and ERK (p-ERK) (**c**) are shown. GAPDH was used for the normalization of the cytosolic proteins. The relative band density of p-Akt in treated (white) and untreated cells (black) were quantified as fold increases compared with the untreated and uninfected control cells (CON). The results shown are representative of three separate experiments. Knockdown of Akt was confirmed by Western blot analysis (**d**). Total RNA was prepared after infection and analyzed by real-time quantitative PCR to estimate amounts of IL-8 transcript in the presence or absence of siAkt (**e**) or LY (**f**). The mRNA expression was normalized to the corresponding GAPDH expression and is shown as the fold increase over control cells. (**g**) IL-8 in the culture medium was assayed by ELISA 5 h later. The amount of secreted IL-8 was expressed as a fold increase compared to the control cells. The results indicate the means and standard deviations for duplicate wells from at least three separate experiments (* *p* < 0.05; N.S.: not significant).

Based on our previous study showing *Salmonella*-induced activation of PI3K/Akt signaling resulted in the inhibition of extracellular signal–regulated kinase (ERK) and consequent suppression of IL-8 expression, [11] we investigated if PI3K/Akt was involved in the negative regulation of IL-8 expression in *Salmonella*-infected SW480 cells by statins. SW480 cells were uninfected or infected with *S.* Typhimurium wild-type strain SL1344, and activation of Akt and ERK was analyzed by Western blotting. The results showed that simvastatin significantly upregulated Akt (p-Akt) activation in *Salmonella*-infected SW480 cells, and ERK was downregulated (Figure 2a), suggesting Akt signaling was involved in the negative regulation by statins. To verify the function of Akt on the regulatory effects of simvastatin on transcriptional and post-transcriptional IL-8 expression in *Salmonella*-infected IECs, Akt siRNA-transfected SW480 cells were treated with or without simvastatin, followed by infection with the wild-type *S.* Typhimurium strain SL1344. Akt knockdown was confirmed by western blotting (Figure 2d). Following the knockdown of Akt, we found that the simvastatin-mediated suppression of *Salmonella*-induced IL-8 mRNA expression and protein production was nearly abolished completely in Akt-silenced cells (Figure 2e,g). Likewise, the suppressive effect of simvastatin on *Salmonella*-induced IL-8 mRNA expression was counteracted in the presence of a PI3K inhibitor (Figure 2f). This suggests the involvement of Akt in simvastatin-mediated negative regulation of IL-8 expression in *Salmonella*-infected IECs.

### 2.3. Simvastatin Up-Regulates VDR mRNA and Protein Expression in Salmonella-Infected SW480 Cells

**Figure 3:**
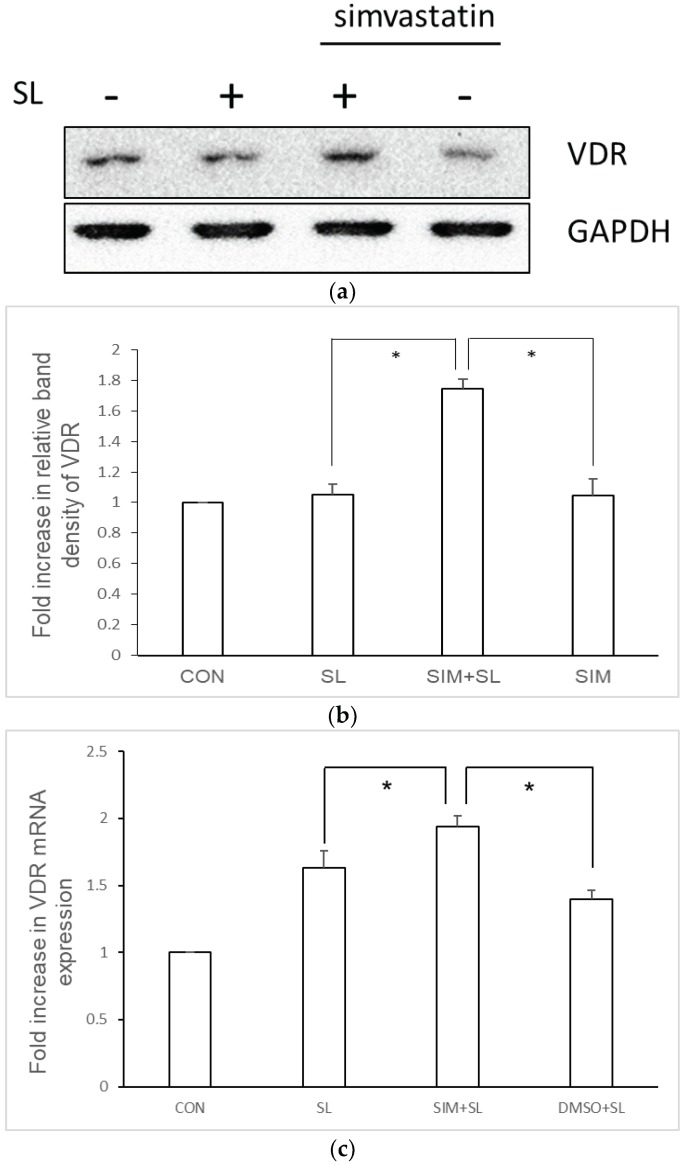
Simvastatin enhances vitamin D receptor (VDR) mRNA and protein expression in *Salmonella*-infected SW480 cells. After treatment with 10 μM simvastatin, SW480 cells were uninfected (CON) or infected with wild-type *S.* Typhimurium strain SL1344 (SL). VDR protein was detected in whole cell lysates by western blotting, which was normalized to GAPDH. Representative immunoblots (**a**) and densitometric quantification of immunoreactive bands (**b**) are shown. The relative band intensities of VDR (**b**) in SW480 cells were quantified as fold increases compared with the control cells. Each value represents the mean ± SD of three independent experiments (* *p* < 0.05). (**c**) Total RNA was extracted after infection and analyzed by real-time quantitative PCR to evaluate the VDR mRNA expression. VDR mRNA expression was normalized to the corresponding GAPDH mRNA expression and is shown as a fold increase over control cells. The results indicate the means and standard deviations for duplicate wells from at least three separate experiments (* *p* < 0.05).

VDR is a transcription factor that plays an important role in regulating the expression of many genes, including antimicrobial peptides [14]. In order to determine if statins upregulate VDR mRNA and protein expression in *Salmonella*-infected IECs, cultured SW480 cells were infected with wild-type *S.* Typhimurium strain SL1344 in the presence or absence of simvastatin. VDR protein and mRNA expressions were analyzed by western blot analysis and RT-PCR, respectively. Figure 3 clearly demonstrates that VDR mRNA and protein expression in SW480 cells was induced by *Salmonella* infection and was upregulated in the presence of simvastatin.

### 2.4. The Involvement of VDR in the Simvastatin-Mediated Negative Regulation of IL-8 Expression in Salmonella-Infected SW480 Cells

**Figure 4:**
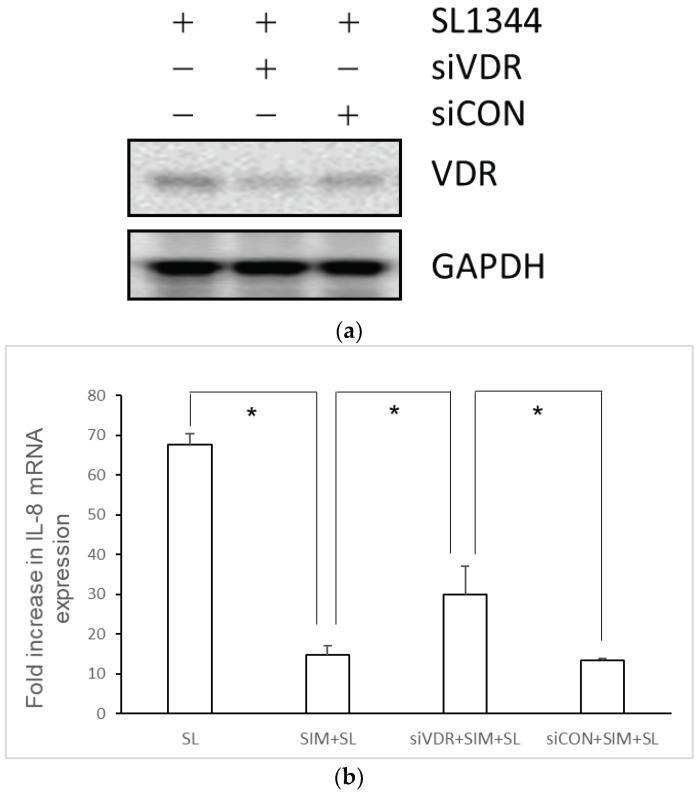
Involvement of vitamin D receptor (VDR) in simvastatin-mediated downregulation of IL-8 mRNA expression in *Salmonella*-infected SW480 cells. SW480 cells were transfected with control siRNA or VDR siRNA (siCON = non-target control siRNA; siVDR = siRNA against VDR) for 48 h. Transfected SW480 cells were treated with 10 μM simvastatin (SIM) prior to wild-type *S.* Typhimurium strain SL1344 infection (SL) for one hour. Knockdown of VDR was demonstrated by western blotting (**a**). Total RNA was extracted and analyzed by real-time quantitative PCR to estimate the levels of IL-8 mRNA (**b**). The amount of IL-8 mRNA produced, normalized to the corresponding amount of GAPDH mRNA, is shown as the fold increase over uninfected control (CON) cells. The results indicate the means and standard deviations for duplicate wells from at least three separate experiments (* *p* < 0.05).

To study the role of VDR in statin-mediated downregulation of IL-8 expression in *Salmonella*-infected IECs, VDR siRNA-transfected SW480 cells were untreated or pre-treated with simvastatin and then infected with wild-type *S.* Typhimurium strain SL1344 for one hour. Total RNA was prepared, reverse transcribed, and analyzed by RT-PCR. Knockdown of VDR was demonstrated by western blotting (Figure 4a). Following VDR knockdown, we observed that simvastatin-mediated downregulation of *Salmonella*-induced IL-8 mRNA expression was rescued in VDR-silenced cells compared to control siRNA-treated cells (Figure 4b). It suggests the involvement of VDR in simvastatin-mediated downregulation of IL-8 expression in *Salmonella*-infected IECs.

### 2.5. The Involvement of VDR in Simvastatin-Mediated Upregulation of NOD2 Expression in Salmonella-Infected SW480 Cells

**Figure 5:**
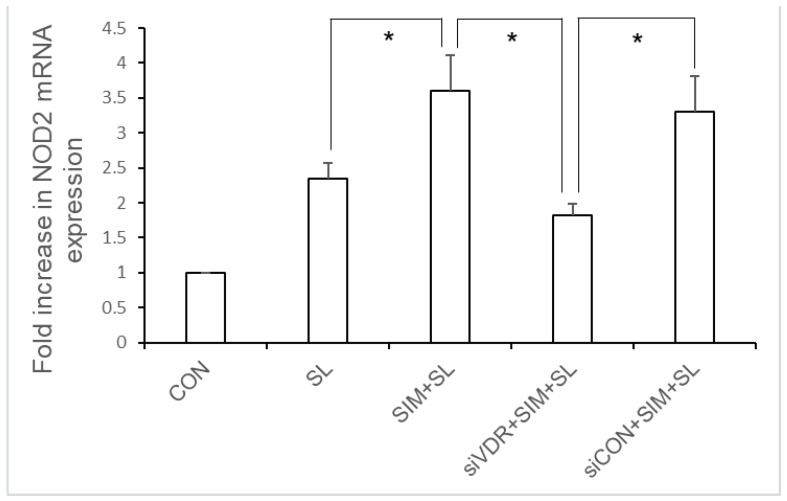
Involvement of vitamin D receptor (VDR) in simvastatin-mediated enhanced NOD2 mRNA expression in *Salmonella*-infected SW480 cells. SW480 cells were transfected with control siRNA or VDR siRNA (siCON = non-target control siRNA; siVDR = siRNA against VDR) for 48 h. Transfected SW480 cells were untreated or pre-treated with 10 μM simvastatin (SIM) prior to wild-type *S.* Typhimurium strain SL1344 infection (SL) for one hour. Total RNA was prepared after the infection and analyzed by real-time quantitative PCR to estimate the levels of NOD2 mRNA. The amount of NOD2 mRNA produced, normalized to the corresponding amount of GAPDH mRNA, is shown as the fold increase over uninfected control (CON) cells. The results indicate the means and standard deviations for duplicate wells from at least three separate experiments (* *p* < 0.05).

To investigate the role of VDR in the statin-mediated enhancement of NOD2 expression in *Salmonella*-infected IECs, VDR siRNA-transfected SW480 cells were untreated or treated with simvastatin and then infected with wild-type *S.* Typhimurium strain SL1344 for one hour. Total RNA was prepared, reverse transcribed, and analyzed by RT-PCR. As shown in a previous study, *Salmonella* infection induced NOD2 mRNA expression in SW480 cells. Following VDR knockdown, we observed that the simvastatin-mediated enhancement of NOD2 mRNA in *Salmonella*-infected SW480 cells was counteracted in VDR-silenced cells but not in control siRNA-treated cells (Figure 5). This suggests the involvement of VDR in the simvastatin-mediated enhancement of NOD2 expression in *Salmonella*-infected IECs.

### 2.6. Involvement of VDR in the Positive Regulation of hBD-2 Expression in Salmonella-Infected SW480 Cells by Simvastatin

**Figure 6:**
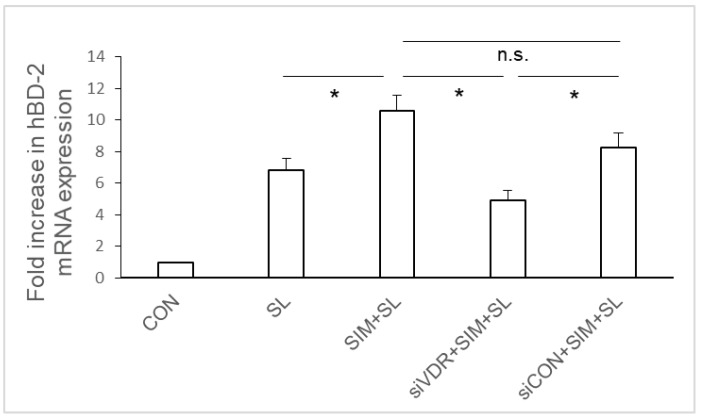
Involvement of VDR in simvastatin-mediated enhanced hBD-2 mRNA expression in *Salmonella*-infected SW480 cells. SW480 cells were transfected with control or VDR siRNA (siCON = non-target control siRNA; siVDR = siRNA against VDR) for 48 h. Transfected SW480 cells were untreated or treated with 10 μM simvastatin (SIM) prior to wild-type *S.* Typhimurium strain SL1344 infection (SL) for one hour. Total RNA was prepared after infection and analyzed by real-time quantitative PCR to estimate the levels of hBD-2 mRNA. The amount of hBD-2 mRNA produced, normalized to the corresponding amount of GAPDH mRNA, is shown as the fold increase over uninfected, control (CON) cells. The results indicate the means and standard deviations for duplicate wells from at least three separate experiments (* *p* < 0.05).

To investigate the role of VDR in the statin-mediated enhancement of hBD-2 expression in *Salmonella*-infected IECs, VDR siRNA-transfected SW480 cells were untreated or treated with simvastatin prior to wild-type *S.* Typhimurium strain SL1344 infection for one hour. Total RNA was prepared, reverse transcribed, and analyzed by RT-PCR. As shown in Figure 6, *Salmonella* infection induced hBD-2 mRNA expression in SW480 cells. Following VDR knockdown, we observed that the simvastatin-mediated enhancement of hBD-2 mRNA in *Salmonella*-infected SW480 cells was counteracted in VDR-silenced cells but not in control siRNA-treated cells (Figure 6). This suggests the involvement of VDR in the simvastatin-mediated upregulation of hBD-2 expression in *Salmonella*-infected IECs.

### 2.7. It Is a General Phenomenon in Intestinal Epithelial Caco-2 Cells

**Figure 7:**
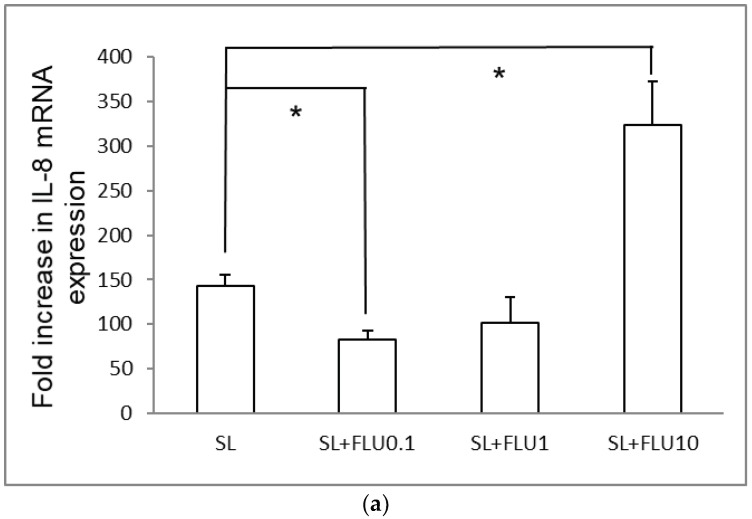

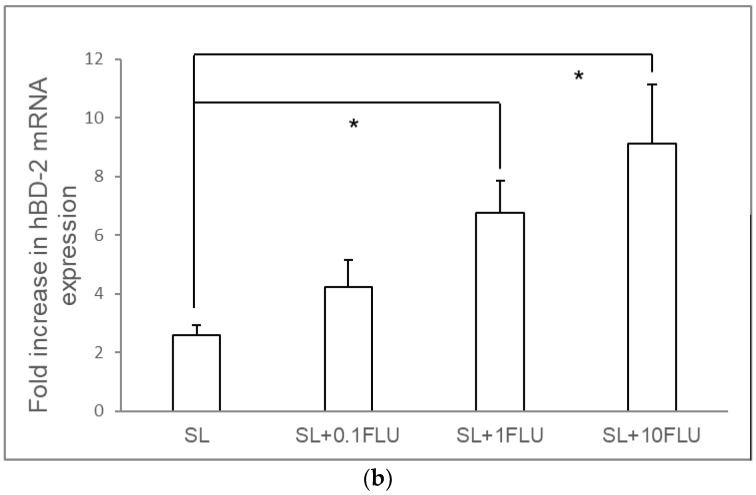
The effect of fluvastatin on IL-8 and hBD-2 mRNA expression levels in *Salmonella*-infected Caco-2 cells. Cultured Caco-2 cells were either uninfected or infected for 1 h with *S.* Typhimurium wild-type strain SL1344 (SL) in the presence or absence of different concentrations of fluvastatin (FLU)(0.1, 1, 10 μM) for 24 h. Total RNA was prepared after infection and analyzed by real-time quantitative PCR to estimate amounts of IL-8 (**a**) and hBD-2 (**b**) transcript. The amount of IL-8 and hBD-2 mRNA produced, normalized to the corresponding amount of GAPDH transcript, is shown as the fold increase over uninfected, control (CON) cells. The results indicate the means and standard deviations for duplicate wells from at least three separate experiments (* *p* < 0.05 compared to *Salmonella* infection only).

**Figure 8:**
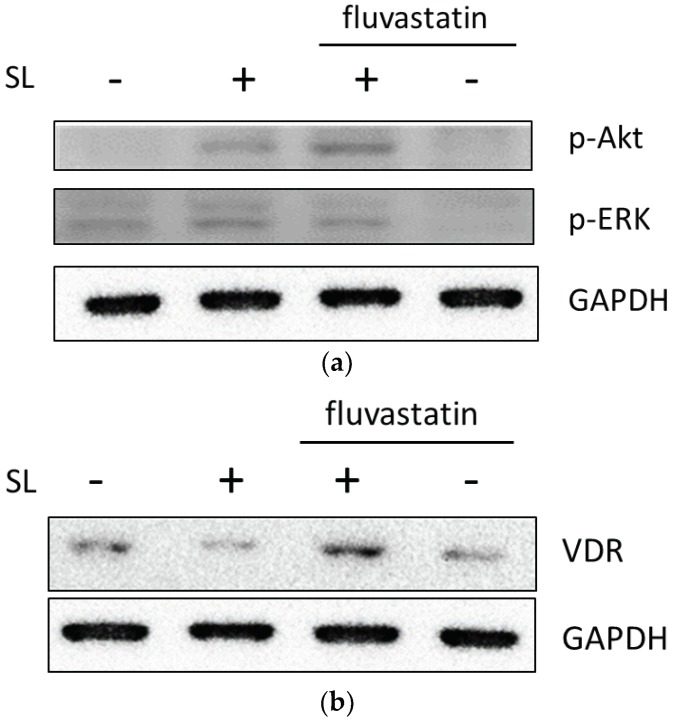
Effect of fluvastatin on the activation of intracellular signals in *Salmonella*-infected Caco-2 cells. Cultured Caco-2 cells were either uninfected or infected for 1 h with *S.* Typhimurium wild-type strain SL1344 (SL) in the presence or absence of 1 μM fluvastatin. Cell lysates were analyzed by immunoblotting with antibodies to phosphorylated (p)-Akt and -ERK (**a**), and VDR (**b**) to evaluate the activation of Akt, ERK, and VDR protein expression. GAPDH was used for the normalization of the cytosolic proteins. Representative immunoblots are shown. The results shown are representative of three separate experiments.

To determine if the above observation was a general phenomenon among different statins and cultured cells, the same experiments were performed using fluvastatin and cultured Caco-2 cells. Caco-2 cells were infected with wild-type *S.* Typhimurium strain SL1344 in the presence or absence of fluvastatin. Total RNA was prepared and reverse transcribed, and proteins were extracted from the cultured cells as described above. RT-PCR and western blotting were performed to evaluate IL-8 and hBD-2 mRNA expressions (Figure 7) and the signaling pathways involved (Figure 8), respectively. The results obtained with fluvastatin in *Salmonella*-infected Caco-2 cells were similar to those observed with simvastatin.

## 3. Discussion

A retrospective case-control study conducted in three hospitals including all hospitalized patients diagnosed with *Clostridium difficile* associated diarrhea (CDAD) [8] revealed that statin use may provide protection against CDAD. Recently, we demonstrated that disruption of membrane cholesterol in IECs may play a protective effect against *Salmonella* invasion [15]. These results suggest that statins may play a protective role in *Salmonella* colitis and its invasion.

Following oral *Salmonella* infection, IECs represent the first defense barrier against the bacteria invading the intestinal tissues. IL-8 released from the infected IECs recruits neutrophils from the circulation into the sub-epithelial region to help contain and eliminate the infection. However, accumulation of neutrophils results in colitis [3]. In contrast, secretion of antimicrobial peptides (human β-defensins) eliminates and defends against the *Salmonella* invasion. We observed that statins upregulate anti-microbial peptide hBD-2 but downregulate pro-inflammatory IL-8 expression in *Salmonella*-infected IECs. This suggests that statins may be used as an alternative therapy for *Salmonella* colitis by defending against and suppressing the pro-inflammatory responses to the luminal bacterial infection. To our knowledge, there have been no reports on how statins affect innate immunity to control *Salmonella* infection. Further in vivo studies to clarify the effect of statins on *Salmonella* colitis and invasiveness are required.

Simvastatin negatively regulates inflammation in chemical-induced experimental colitis [16]. Our study demonstrates that statins at lower doses suppress IL-8 expression in *Salmonella*-infected IECs. Clinically, acute gastroenteritis with pathogens such as *Salmonella* is followed by increased risk of inflammatory bowel disease (IBD) [17]. These observations suggest that simvastatin has therapeutic potential in human infectious diseases and IBD. In contrast, increased IL-8 production within the intestine of patients with active IBD may contribute to neutrophil activation, and may therefore initiate or perpetuate the disease [18]. Our study observed that statins at high doses enhanced *Salmonella*-induced IL-8 expression, which could be hazardous for patients with IBD complicated by intracellular bacterial infection, such as *Salmonellosis*. Although colitis is a rare complication of simvastatin treatment, development of ulcerative colitis has been reported to be an adverse reaction to simvastatin [19], which, despite drug withdrawal, has proved fatal. This supports the possible complication of statin overdose and provides its underlying mechanisms. In fact, excessive lowering of cholesterol may also enhance vulnerability to infection. A cohort study observed an inverse association between serum total cholesterol and in-hospital incidence of infectious diseases [20], though the association is weak and not entirely consistent.

Antibiotics, such as clarithromycin or erythromycin, may inhibit the metabolism of statins and increase statin concentration in the blood, which can cause muscle or kidney damage, and even death. However, these adverse reactions are rare. In contrast, an increasing number of studies are emerging which detail the attenuation of bacterial growth as well as the in vitro and in vivo virulence by statin treatment [21]. Although the effect of statins is hindered at high concentrations—which includes interactions with antibiotics [22], gut dysbiosis-associated metabolic effects [23], impact on drug-microbiome interactions [24], and antibiotic-like side effects and antibiotic resistance—this hindrance may be overcome using subinhibitory concentrations of statins in combination with existing antibiotics. Thus, further randomized control trial and prospective studies have been recommended. The most improvements may be achieved by matching particular statins with particular infecting pathogens.

Accumulating evidence has also shown that VDR is critical for the control of innate immunity in the gastrointestinal tract and in experimental IBD [25,26]. VDR knockout mice were hyper-responsive to exogenously injected lipopolysaccharide, accompanied by high colonic expression of inflammatory cytokines and increased bacterial growth in the peritoneal exudates of moribund mice [25]. In melanoma cell lines, VDR mediates the repression of TNF-α-induced IL-8 promoter activity by calcitriol [27]. We observed that statins stimulated VDR mRNA and protein expressions, and VDR knockdown abolished the suppressive effect of statins on the IL-8 expression in *Salmonella*-infected IECs. This suggests the involvement of VDR in statin-mediated suppression of inflammation during *Salmonella* colitis. Another possibility is the bactericidal effect of statins on *Salmonella* extracellularly. However, in the review by Hennessy et al. [21], they have presented the minimum inhibitory concentration of statins against certain Gram-positive and Gram-negative bacteria. They reported that simvastatin did not inhibit the growth of a range of Gram-negative pathogens including *Salmonella enterica* serovar Typhimurium, as used in our study. This reinsures the justification of our experiments because the cellular innate immunity we investigated seems not to be influenced by the direct antimicrobial effect. Additionally, the differences in the production of IL-8 could be independent of the bacterial load.

The beneficial effects of the statin therapy were mediated via VDR activation by this group of drugs because a striking similarity between the benefits of vitamin D and statin therapy was observed [28]. Each of the statins has a different affinity for this key receptor. Only simvastatin was able to exert a direct effect on VDR at a normal therapeutic dose, although lovastatin was effective at a higher therapeutic dose [29]. Accumulating evidence suggests that VDR binds to PI3K, an upstream effector of AKT, and activates the PI3K-AKT signaling pathway in cancer cells [30]. PI3K/AKT-dependent inhibition of ERK has an anti-inflammatory effect on IL-8 expression in *Salmonella-*infected IECs [11]. This supports our observation that statins suppressed pro-inflammatory IL-8 expression in *Salmonella*-infected IECs via the VDR-PI3K-AKT pathways. However, high-dose statins on the other hand may deplete the membrane cholesterol in *Salmonella*-infected IECs and lead to upregulation of IL-8 expression via inhibition of Akt activation [15]. This effect may overcome the effect of VDR that enhances Akt activation and results in the suppression of IL-8 expression.

Additionally, PI3K/Akt mediates anti-apoptotic effects in IECs to enhance their resistance to injury [31]. The intact mucosal barrier prevents bacterial invasion and thus reduces mucosal inflammation while excessive apoptosis causes focal disruption of the mucosal barrier. Furthermore, PI3K/Akt is a critical regulatory signaling pathway in the immunological defense system against sepsis [32]. Several investigations [32] support a beneficial role for PI3K-mediated effects in combating sepsis through ameliorating the host response to septic injury. We observed that a lower dose of statins enhanced the activation of Akt, which may result in anti-apoptotic effects and resistance to mucosal injury, thus leading to the prevention of bacterial invasion. Furthermore, increased IEC apoptosis has been reported in patients with IBD [33] as well as in murine models of colitis [34]. VDR plays a critical role in mucosal barrier homeostasis by maintaining the integrity of junction complexes and the repairing ability of the colonic epithelium [35]. This suggests that statins might exert anti-apoptotic effects and maintain intestinal mucosal integrity via VDR and Akt expression.

Statin treatment can lower bacterial burden during *S. enterica* [36] and *Chlamydia pneumoniae* [37] infection in mice, but the mechanism underlying the antimicrobial activity of statins remains inconclusive. We demonstrated that statins enhanced hBD-2 expression in *Salmonella*-infected IECs, and may lead to the direct elimination of mucosal bacteria, which can explain the protective effects of statins against the invasiveness of *Salmonella* infections [38]. The observation that NOD2 is a VDR target gene [13] and that NOD2 mediates induction of hBD-2 in epithelial cells [12] suggests that statins may mediate hBD-2 expression in *Salmonella*-infected IECs via VDR. We observed that statins enhanced VDR mRNA and protein expressions and subsequent NOD2 expression, resulting in enhancement of hBD-2 expression. However, the mechanisms how statins upregulate VDR expression remain unknown. Two reports have indicated that statins increased gene expression through the inhibition of histone deacetylase activity [39], and that sulforaphane, a dietary histone deacetylase inhibitor, induces VDR-mediated hBD-2 expression in IECs via induction of VDR expression [40], suggesting that statins could also increase VDR-mediated gene expression through the inhibition of histone deacetylase activity. Moreover, it is likely that VDR is upregulated by activation of Toll-like receptors [41] or a sensitization mechanism affecting the epithelial cells could also be involved.

## 4. Methods and Materials

### 4.1. Bacterial Strains

The wild-type *Salmonella enterica* serovar Typhimurium (*S.* Typhimurium) strain SL1344 was used in this study, and the *Salmonella* inoculum was prepared as described previously [42,43].

### 4.2. Cell Culture and Infection

Caco-2 (passage 20–23) and SW480 cells (passage 52–63) were purchased from the American Type Culture Collection (Manassas, VA, USA) and were grown as previously reported to 50−75% confluency [42,43]. Aliquots of bacterial suspension (25 or 50 μL; 1–2 × 10^8^ bacteria cells) were prepared as previous described [42,43] and used to infect the cultured cells. The bacterial inoculum was adjusted to a bacteria-to-cell ratio of 200:1.

### 4.3. Reagents

Standard laboratory reagents were purchased from Sigma-Aldrich (St. Louis, MO, USA) or Fisher Scientific (Pittsburgh, PA, USA). The standard stock solution of simvastatin and fluvastatin (Sigma-Aldrich, St. Louis, MO, USA) was prepared in dimethyl sulfoxide (DMSO) and water, respectively, stored at −20 °C, and further adjusted to the appropriate concentration with cell culture medium immediately before use.

### 4.4. Cytokine Assays

IL-8 protein secreted in the culture supernatants were measured by ELISA (BD Biosciences Pharmingen, San Diego, CA, USA), according to the manufacturer’s instructions and modified as described previously [43].

### 4.5. Cell Fractionation

Extracted protein fractions from untreated and treated cultured cells were prepared by Nuclear/cytosolic Fractionation or Membrane Protein Extraction Kit (ThermoFisher Scientific, Waltham, MA, USA) as recommended by the manufacturer or modified as in previous studies [42,43]. The protein concentrations in the cell fractions were determined using a Bio-Rad assay kit.

### 4.6. Western Blotting

Equal amounts of extracted protein from the fractionation of cultured cells were separated by SDS-PAGE and then transferred to nitrocellulose membranes by semi-dry blotting as previously described [42,43]. After blocking the membranes with 5% non-fat dry milk, proteins expressions were analyzed with antibodies to either phosphorylated Akt (#4058, 1:1000) or ERK (#9101, 1:1000) (Cell Signaling Technology, Danvers, MA, USA) or VDR (#12550, 1:1000) (Cell Signaling Technology, Danvers, MA, USA), and then developed with horseradish peroxidase-conjugated secondary antibodies and enhanced chemiluminescence reagents (ThermoFisher Scientific, Waltham, MA, USA). Appropriate exposures to X-ray film were performed, and the membranes were then stripped and re-probed with antibodies to total GAPDH (sc-137179, 1:1000) (Santa Cruz Biotechnology, Dallas, TX, USA) as appropriate.

### 4.7. RNA Isolation and cDNA Synthesis

Total RNA was extracted from cultured cells using Trizol reagent (ThermoFisher Scientific, Waltham, MA, USA), following the manufacturer’s directions. The cDNA generated by reverse transcription was amplified using PCR as described previously [42,43].

### 4.8. Real-Time Reverse Transcription PCR

Real-time reverse transcription-PCR analyses were performed in a fluorescence temperature cycler (LightCycler; Roche Diagnostics, Indianapolis, IN, USA) as described previously [42,43] to determine the IL-8, hBD2, and NOD2 mRNA expression levels using the comparative threshold cycle (ΔΔ*C*t) method of relative quantitation.

### 4.9. RNA Interference (RNAi) in Cultured Cells

RNAi experiments in cultured cells were performed as previously reported. Cultured cells were transfected according to the manufacturer’s protocol, which was modified in our laboratory [42,44,45]. Briefly, cells were transfected with protein kinase B (Akt) siRNA (Invitrogen-Thermo Fisher Scientific, Waltham, MA, USA, Cat #1299001), VDR siRNA (Ambion-Thermo Fisher Scientific, CA, USA, Cat #4390824), or control siRNA duplex (Santa Cruz Biotechnology, Dallas, TX, USA) by lipofectamine RNAiMAX (Invitrogen-Thermo Fisher Scientific, Waltham, MA, USA). The efficiency of silencing was confirmed by western blot analysis. After a 48 to 72-h incubation at 37 °C, the cells were left untreated or treated by statins before infection with bacteria. The cells were then lysed and the RNA or proteins were extracted on ice for analysis.

### 4.10. Cell Viability and Morphologic Features

Representative cell populations from each condition were examined by light microscopy. No significant morphological change was observed under experimental condition. Cell viability was also confirmed by trypan blue exclusion.

### 4.11. Statistical Analysis

All above experiments were carried out at least in triplicate with similar results. Statistical analysis was performed using the paired Student’s *t*-test for comparison of two means and ANOVA for three or more means (SPSS Statistics; IBM, Armonk, NY, USA). *p* values < 0.05 were considered significant.

## 5. Conclusions

In conclusion, we observed the differential regulation of statins on pro-inflammatory chemokine IL-8 and antimicrobial peptide hBD-2 in *Salmonella*-infected IECs via the expression of different proteins and signaling pathways. Downregulation of the pro-inflammatory response by statins prevents the detrimental effects of overwhelming inflammation in the host, whereas enhanced activity of antimicrobial peptides in the IECs protects the host against infection. Thus, the effects of statins on innate immunity in IECs may provide an alternative or combination therapy for *Salmonella* colitis and IBD. However, well-controlled clinical trials are needed for the administration of statins as an adjunctive treatment in *Salmonella* infection or IBD.
